# The effects of lncRNA MALAT1 on proliferation, invasion and migration in colorectal cancer through regulating *SOX9*

**DOI:** 10.1186/s10020-018-0050-5

**Published:** 2018-10-03

**Authors:** Yuanlin Xu, Xihong Zhang, Xiufeng Hu, Wenping Zhou, Peipei Zhang, Jiuyang Zhang, Shujun Yang, Yanyan Liu

**Affiliations:** 1 Department of Lymphatic Comprehensive Internal Medicine, Affiliated Cancer Hospital of Zhengzhou University, No.127 Dongming Road, Zhengzhou, 450001 Henan China; 2 Department of Gynaecology and Obstetric, Pepole’s Hospital of Henan University of Chinese Medicine (Pepole’s Hospital of Zhengzhou), Zhengzhou, 450003 Henan China; 3 Department of Respiratory, Affiliated Cancer Hospital of Zhengzhou University, Zhengzhou, 450001 Henan China

**Keywords:** lncRNA MALAT1, miR-145, *SOX9*, Colorectal cancer

## Abstract

**Background:**

For the study, we determine the potential biomarkers and uncover the regulatory mechanisms of lncRNA MALAT1 / miR-145 / *SOX9* axis on the abilities of cell growth and cell metastasis of colorectal cancer.

**Methods:**

Previously published dataset GSE18105 from GEO database was used for microarray analysis to identify differential-expressed lncRNAs and mRNAs. The miRNA which had targeted relationships with both lncRNA and mRNA was predicted using miRCode and Targetscan. The association between lncRNA and miRNA, miRNA and mRNA was verified using dual-luciferase reporter assay.

Expression levels of lncRNA MALAT1, miR-145 and *SOX9* were examined by quantitative RT-PCR analysis. The cell viability of two cancer cell lines was compared by CCK-8 assay. Colony formation was hired to detected cell proliferation. The cell cycle distribution and apoptotic cell rate were conducted by flow cytometry assay. Wound healing as well as transwell assay were compare the cell migration and cell invasion respectively among groups. The effect of MALAT1 on colorectal cancer in vivo was constructed by xenograft model.

**Results:**

Significantly dysregulated lncRNAs and mRNAs were identified by microarray analysis. By experimental verification, MALAT1 and *SOX9* were expressed in a high percentage of colorectal cancer tumors and cells, while miR-145 was in a low expression. We also identified miR-145 as a target of MALAT1 and *SOX9*. MALAT1 played a role in regulating cancer process by functioning as a competing endogenous RNA. Silencing MALAT1 could effectively decrease the expression level of *SOX9*, thus suppress cell viability and metastasis. Down-regulated MALAT1 could induce resistance of G1 phase in cell cycle, and facilitation of colorectal cancer cell apoptosis. Nude mice injected with cells transfected with si-MALAT1 had smaller tumor on size and weight.

**Conclusions:**

The regulatory function of lncRNA MALAT1 / miR-145 / *SOX9* axis was revealed in colorectal cancer based on bioinformatics analysis. LncRNA MALAT1 could facilitate colorectal cancer cell proliferation, invasion and migration by down-regulating miR-145 and up-regulating *SOX9*. LncRNA MALAT1 could suppress cell cycle and apoptosis through MALAT1 / miR-145 / *SOX9* axis.

## Background

Colorectal cancer is at the third place which leads to death worldwide (Siegel et al. 2014), with a high capacity for tumor invasion and migration. The development of colorectal cancer from normal epithelial cells to malignant carcinomas is considered to be a polystage process involving disruption of cell survival mechanisms, such as cell proliferation, differentiation and apoptosis (Rupnarain et al. 2004). Although metastasis has been recognized as the most deadly attributes of colorectal tumors (Rasool et al. 2013), the molecular underpinnings of colorectal cancer proliferation still remain incompletely understood. Accordingly, the identification of novel molecules which are differentially expressed in colorectal cancer may afford insights into the mechanisms involved.

Long non-coding RNAs (LncRNAs), greater than 200 nucleotides in length, are arbitrarily defined as RNA molecules that do not contain any apparent protein-coding potential determined largely via bioinformatics (Bergmann and Spector 2014). Although lncRNAs were previously regarded as spurious transcriptional noise on account of RNA polymerase II low specificity (Struhl 2007), they were recently elucidated to involve in the modulation of gene expression through dosage compensation, nuclear organization, transcriptional regulation and imprinting (Bonasio and Shiekhattar 2014). MALAT-1, metastasis associated lung adenocarcinoma transcript-1, named after its function that was initially discovered, is transcribed from the nuclear-enriched transcript 2 (NEAT2) for about 8.1 kb in length (Ji et al. 2003). MALAT-1 was later notarized as a transcript with abundant nuclear enrichment (Hutchinson et al. 2007) and expressed in the lungs, nerve system, pancreas and other human organs (Hutchinson et al. 2007). High expression of MALAT1 has been reported in multiple cancers. For instance, up-regulation of long non-coding RNA MALAT-1 conferred poor prognosis and influenced cell proliferation and apoptosis in acute monocytic leukemia (Huang et al. 2017). In addition, it indicated a poor prognosis and induced migration and carcinoma growth in non-small cell lung cancer (Schmidt et al. 2011). In lung adenocarcinoma cells, MALAT-1 was also reported enhancing cell motility by affecting the expression quantity of motility-related genes (Tano et al. 2010). Besides, over-expression of MALAT-1 was found in colorectal cancer, (Yang et al. 2015). With the enhancement of kinase (PRKA) anchor protein 9 (AKAP-9) expression, MALAT-1 could accelerate cell proliferation, migration and invasion in vitro (Yang et al. 2015), which implied that MALAT1 acted as a potential role in the progression of colorectal cancer.

*Sex-determining region Y (SRY)-box 9 (SOX9)*, a transcription factor, was critical for cancer progression and reported as oncogene (Qian et al. 2017). Recent studies presented that uncontrolled expression of *SOX9* was found in many kinds of cancers, such as glioma (Liu et al. 2016a), lung cancer (Li et al. 2017), colorectal cancer (Carrasco-Garcia et al. 2016) and so on. More importantly, *SOX9* with up-regulated expression was indicated poor prognosis in colorectal cancer, glioma and lung cancer (Liu et al. 2017; Bruun et al. 2014; Zhou et al. 2012). *SOX9* over-expressed in colorectal cancer was reported by (Javier et al. 2016; Montorsi et al. 2016 and Shi et al. 2015). However, the mechanisms underlying *SOX9* mediated tumorigenesis remain elusive.

MicroRNAs (miRNAs) were small endogenous non-coding RNA molecules which played a crucial role in regulating gene expression by interaction of specific transcripts (Yang et al. 2017). MiR-145 was verified to suppress tumor development and found decreased in colorectal cancer (Sheng et al. 2017). In the further studies, miR-145 has been demonstrated that it took part in the progression of colorectal cancer by controlling a series of related gene expressions participated in oncogenesis and metastasis (Wang et al. 2016; Li et al. 2016). As for upstream regulation, Arun et al. discovered that MALAT1 regulated miR-145 in gastric cancer as a competing endogenous RNA (ceRNA) (Arun et al. 2018). Nevertheless, molecular mechanisms of the ceRNA axis of MATAL1 and miR-145 modulating colorectal cancer process were rarely explored.

Taken together, to make an investigation on the critical function and mechanism of MALAT1/miR-145/*SOX9* in colorectal cancer, the expression and the correlation among MALAT1, miR-145 and *SOX9* were determined. Then we investigated the influence on cell proliferation, invasion, migration, cell cycle and apoptosis of colorectal cancer through MALAT1 / miR-145 / *SOX9*. Better understandings of the role of competing endogenous RNA MALAT1, miR-145 and *SOX9* will have translational potential for early diagnosis and may lead to the progress of novel treatment strategy against malignant colorectal tumor.

## Methods

### Human tissue samples

Forty pairs of freshly frozen colorectal tumors and corresponding normal mucous tissue (5 cm away from the cancer lesions) were collected from colorectal cancer patients who underwent colorectal resection at Affiliated Cancer Hospital of Zhengzhou University. Each AJCC classification (I-IV) had ten cases. Tissue samples were stored at a low-temperature environment until further use. Tumor samples contained more than 80% of tumor cells. Specimens are handled with very close attention to maintaining integrity and isolation. For this study tissues were held briefly at − 80 °C during frozen sectioning, using 100% ethanol to clean the blade between all samples. For each of the 40 subjects in our study, one tumor section and one matched adjacent tissue were analyzed, totaling 80 samples. The pathological diagnosis of colorectal cancer specimens and confirmation of the adjacent normal intestinal mucosa specimens were performed by at least two pathologists. No pre-operative chemotherapy or radiotherapy treatments were taken on patients. The Clinical Research Ethics Committee of Affiliated Cancer Hospital of Zhengzhou University approved the research protocols. All patients were signed the informed consents.

### Bioinformatics analysis

GSE418105 dataset containing lncRNA and mRNA expression profiles were retrieved from the Gene Expression Omnibus (GEO) database (http://www.ncbi.nlm.nih.gov/geo/). LncRNAs and mRNAs differentially expressed in colorectal cancer tissues from normal tissues were obtained through microarray analysis. Microarray type used in the microarray analysis was HG-U133_Plus_2, obtained from GEO. A total of 85 samples were adopted. The target relationships between miRNA and the selected lncRNA and mRNA were found using miRCode and Targetscan (Whitehead Institute, Cambridge, MA, USA). GO analysis was performed to explore the relationship between differential genes and molecular function, and to show the gene enrichment in different biological processes like migration, apoptosis, cell cycle and so on.

### Cell culture

Human normal colorectal epithelial cell line NCM-460 and cell lines for colorectal cancer DLD-1, HT-29 (BNCC, Beijing, China) were cultured in RPMI-1640 medium (Haoranbio, Shanghai, China) supplemented with 10% fetal bovine serum (FBS; Hyclone, Logan, UT, USA; 100 mg/L). A humidified incubator containing 5% CO_2_ was used to incubate cells at 37 °C.

### Vector construction

Small interference RNA (siRNA) and negative control (Invitrogen, Carlsbad, CA, USA) was hired to knockdown MALAT1, *SOX9* and controls. The following siRNAs were used to target human MALAT1: sense: 5’-CACAGGGAAAGCGAGTGGTTGGTAA-3′ and antisense: 5’-TTACCAACCACTCGCTTTCCCTGTG-3′. The following siRNAs were used to target human *SOX9*: sense: 5’-GGGUCUCUUCUCGCUCUCGTT-3′ and antisense: 5’-AACGAGAGCGAGAAGAGACCC-3′. The sequence of the negative control was: 5’-TTCTCCGAACGTGTCACGT-3′. The full-length *SOX9* was inserted into the clone pcDNA3.1 (−) for further use. The restriction enzyme cutting sites were *Not*I and *EcoRI*. The clone pGCSIL-GFP slow virus expression vector (GeneChem, Shanghai, China) was inserted.

### Cell transfection

In logarithmic phase DLD-1 and HT-29 cells were obtained and resuspended in RPMI-1640 medium (Hyclone, South Logan, UT, USA) supplemented with 10% FBS (Hyclone, Logan, UT, USA). Cells with the destiny of 6 × 10^5^/well were seeded in 24-well plates at for 24 h before transfection. Adherent cells were transfected with interference sequences and pGCSIL-GFP recombinant vector using Lipofectamine 2000 reagent (Invitrogen). 48 h later, transfection efficiency was tested.

### qRT-PCR

Total RNA, including the small RNA fraction, was extracted from collected tissues and each cell lines with TRIZOL reagent (Invitrogen). Next, reverse transcription reactions were carried out with PrimeScript RT Reagent kit (Takara, Dalian, China), and quantitative real-time PCR (qRT-PCR) was performed using SYBR Premix DimerEraser (Takara, Dalian, China) and the Step One Plus Real-time PCR system (Applied Biosystems, Foster City, CA, USA). Primers (Shanghai Generay Biotechnology Co., Ltd.) were listed in Table 1. Quantitative PCR was performed using TaqMan 2×universal master mix (Applied Biosystems, Foster City, CA, USA) and using the following cycle conditions: 95 °C for 30 s, 95 °C for 5 s and 40 cycles of 62 °C for 40 s. GAPDH was employed as the endogenous control. The ${2}^{-\Delta  \Delta  {C}_t}$ method was used to calculate the relative expression.

**Table 1:** The sequences of primers

cDNA	Sequences (5′-3′)
MALAT1 (forward)	5’-TGCGAGTTGTTCTCCGTCTA-3’
MALAT1 (reverse)	5’-TATCTGCGGTTTCCTCAAGC-3’
SOX9 (forward)	5’-TGCAGGAGGAGAAGAGAAGG-3’
SOX9 (reverse)	5’-GTGGCCAGTTCACAGCTGC-3’
GAPDH (forward)	5’-CTGGGCTACACTGAGCACC-3’
GAPDH (reverse)	5’-AAGTGGTCGTTGAGGGCAATG-3’

### Western blot

NCM-460, DLD-1 and HT-29 cells together with the corresponding transfection cells in logarithmic phase were obtained. Proteins from tissue samples and colorectal cancer cell lines were extracted with lysis buffer. The BCA Protein Assay kit (both from Beyotime Institute of Biotechnology, Haimen, Jiangsu, China) was utilized to determine the protein concentration. Protein lysate was separated on 12% SDS-PAGE gels, and then transferred onto PVDF membranes (Millipore, Billerica, MA, USA). After that, 5% non-fat dried milk was made use of blocking the membrane. Next, membranes were incubated with rabbit anti *SOX9* monoclonal antibody (ab6721; Abcam, Cambridge, MA, USA) or GAPDH (ProteinTech, Chicago, IL, USA) overnight, then washed with Tris-buffered saline with Tween (TBST) four times, and cultured with horse radish peroxidase (HRP) labeled goat anti-rabbit secondary antibody for 2 h. Enhanced chemiluminescence (ECL) reagent (Pierce, Rockford, IL, USA) was taken to reveal protein bands.

### Dual-luciferase reporter assay

Wild type MALAT1 and wild type *SOX9* were connected to pmiR-RB-REPORORT™ Vector (Ribobio, Guangzhou, China). MUT-MALAT1 and MUT-*SOX9* were used as the control. The mutation vectors of MALAT1 3’UTR and *SOX9* 3’UTR were constructed and the recombinant plasmid sequences were identified. The restriction enzyme cutting sites were *Xho*Ι and *Not*Ι. Mutation was performed using QuikChange II XL Site-Directed Mutagenesis Kit (Strategene, La Jolla, CA, USA). For the luciferase assay, NCM-460 cells were grown in DMEM medium (Invitrogen) and collected after digestion. Several 96-well plates were employed to seed cells. Plasmid DNA (0.2 μg) and Fugene HD (0.3 μl) were added into miR mimics after mixing. Each group had three compound holes. 0.15 μg sensor reporter genes and 0.9 μl FugeneHD were diluted by 30 μl Opti-MEM medium. Groups included NC-MUT-MALAT1, NC-WT-MALAT1, miR-mimics-MUT-MALAT1, miR-mimics-WT-MALAT1, NC-MUT-*SOX9,* NC-WT-*SOX9*, miR-mimics-MUT-*SOX9* and miR-mimics-WT- *SOX9*. After cells being transfected for 48 h, lysis buffer was diluted by double distilled water and the medium was abandoned. 80 μl lysis buffer was added into each hole and centrifuged for 1 h. Sediment was collected and added into non-transparent 96-well plates with firefly luciferase and sea cucumber luciferin substrate. Luciferase activities were gauged by a Dual-Luciferase Reporter Assay Kit (Promega, Madison, WI, USA).

### Scratch wound healing assay

Scratch wound healing assay was performed for analysis of cell migration in vitro. Briefly, DLD-1 and HT-29 cells transfected with si-MALAT1, miR-145 inhibitor, si-MALAT1 + miR-145 inhibitor, si-*SOX9*, pcDNA3.1-*SOX9*, si-*SOX9* + miR-145 inhibitor and negative control miRNA or mRNA. In order to further understand the effect of MALAT1 / miR-145 / *SOX9* axis on cell migration, wound healing assays were also conducted in the presence of the cell proliferation inhibitor mitomycin C (MMC, Sigma-Aldrich, St. Louis, MO). The DLD-1 and HT-29 cells were cultured in 6-well plates (5 × 10^5^/well) and incubated overnight. Culture inserts were removed after appropriate cell attachment and washed twice using PBS. Afterwards, cells were added in the DMEM medium with 10% FBS. At 0 and 24 h after scratch would formation, images were obtained using an inverted microscope (Nikon, Tokyo, Japan) at a magnification of 200× and measured by Image-Pro Plus software (Media Cybernetics, Inc., Rockville, MD, USA).

### Colony formation assay

DLD-1 and HT-29 cells transfected with si-MALAT1, miR-145 inhibitor, si-MALAT1 + miR-145 inhibitor, si-*SOX9*, pcDNA3.1-*SOX9*, si-*SOX9* + miR-145 inhibitor were obtained. Cells were digested by trypsin solution and then added into RPMI1640 (Hyclone, South Logan, UT, USA) medium. The cell suspension was seeded into a petri dish with appropriate concentration. Petri dishes were gently vibrated to let the cells spread evenly and placed at a constant temperature to cultivate cells for 14–21 days at a routine culture environment. The culture was terminated when visible cells were observed. PBS was used to wash cells and 10% methanol and 10% acetic acid were utilized to fix cells. Then 0.4% crystal violet was used to stain cells. Then the staining was removed. The colony numbers were counted using ColCounte colony counter (Oxford Optronix Ltd., Abingdon, UK). Sensitivity was set to cell number > 50. The efficiency of plating (PE) was calculated by the following formula: PE = the number of colonies/the number of plated cells. PE was standardized to the blank group. The following formula was taken to calculate survival rate (SF): SF = the number of colonies/the number of plated cells × PE.

### Cell invasion assay by transwell

For the invasion assay, DLD-1 and HT-29 cells were put into the upper chamber of each well of a 24-well transwell polycarbonate membrane (8-mm pore size; Costar, Cambridge, MA, USA) coated with Matrigel (BD Biosciences, San Jose, CA, USA). Medium containing 10% FBS, served as a chemoattractant, was put into the lower chambers. After wells were incubated for 16 h at 37 °C, the surface of cells on the upper membrane were removed. Cells were then fixed and stained with 0.2% crystal violet solution for 30 min. An inverted microscope (IX71; Olympus, Japan, Toyoko) was applied to count (five high-power fields per chamber) the invading cells attached the adaxial surface of the filter were counted.

### Cell proliferation assay by CCK-8

Cell Counting Kit-8 (CCK-8) was performed to test cell viability. Cells were put into 96-well plates at l × l0^4^/well in complete medium and cultured for 24 h. The medium was then replaced with medium containing 10% FBS, with or without treatment. After incubation, 10 μL of CCK-8 reagent was inserted to each well, and the plates were further incubated for 4 h. Proliferation rates were determined every 12 h after transfection. The spectrophotometric absorbance at 450 nm was measured for each sample. All the experiments were performed at least three times and the mean was calculated.

### Cell cycle and apoptosis assays by flow cytometry

For cycle analysis, the test was performed using the cell cycle kit (#A10798; Invitrogen, Carlsbad, CA, USA). Cells were cultured in 6-well plates at the destiny of approximate 5 × l0^4^ cells/well. Cells with 72 h transfection were digested with pancreatin and centrifuged for about 5 min at 1200 rpm. After mediums were abandoned, the cells were washed and centrifuged for 5 min at 1000 rpm and then collected. After being fixed in the refrigerator with 1 ml pre-cooled 70% alcohol at 4 °C overnight, cells were washed twice with PBS and incubated with 100 μl of RnaseA in the dark at 37 °C for 30 min. The flow cytometry was conducted after cells were treated with 50 μl PI for 1 h in darkness.

For apoptosis analysis, cells were put in 6-well plates at the destiny of approximate 5 × l0^4^/well. Cells transfected for 48 h were collected, washed twice with cold PBS and re-suspended in binding buffer with a density of 1 × 10^6^/mL. Cells were next stained with FITC (BioVision, San Francisco, CA, USA) and PE (Beyotime). The signal was acquired by a FACS Calibur flow cytometer (BD Biosciences) and was analyzed with FACS Diva software (BD Biosciences). Experiments were conducted in triplicate.

### Xenograft model

DLD-1 and HT-29 cells were transfected with si-NC and si-MALAT1. 2 × 10^5^ cells were injected into female BALB/c-nu nude mice (weighing 15–20 g, Department of Laboratory Animal, Fudan University). Each group had 5 mice. Tumor size of the mice was measured and calculated at the interval of 7 days. After 28 days, the mice were decapitated and the tumors were excised. The growth of tumors in vivo was visualized and imagined using GFP imaging system (Lighttools, Encinitas, Canada), and the tumor volume was measured with vernier caliper. Animal experiments were approved by the Institutional Animal Care and Use Committee of Affiliated Cancer Hospital of Zhengzhou University and performed following the Institutional Guidelines and Protocols.

### Statistical analysis

Statistical analysis was using SPSS 20.0 and GraphPad Prism 6.0 (GraphPad Software, San Diego, CA). Data were presented in the form of the “mean ± standard”. Student’s t-test was used to assess differences between two groups. One-way analysis of variance (ANOVA) was used to compare differences among three groups or more. Rank-sum test was used to evaluate heterogeneity of variance. All experiments were with 3 repetitions for each group. *P* < 0.05 was considered statistically significant.

## Results

### MALAT1 and *SOX9* were high expressed in colorectal cancer tissues

**Fig. 1:**
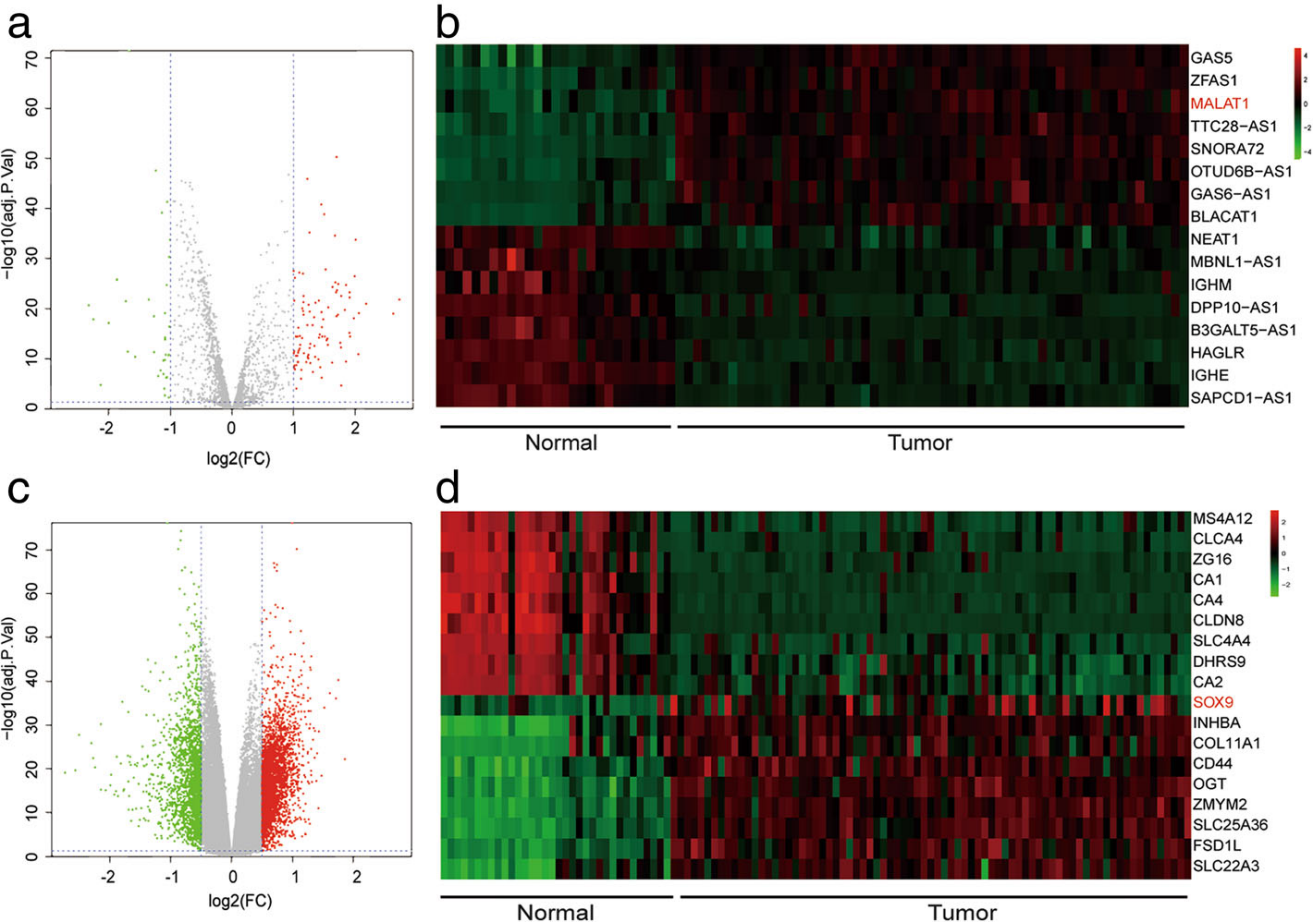
Microarray analysis and verification **a** Volcano plot of lncRNAs. **b** Heat map of lncRNAs. **c** Volcano plot of mRNAs. **d** Heat map of mRNAs

**Fig. 2:**
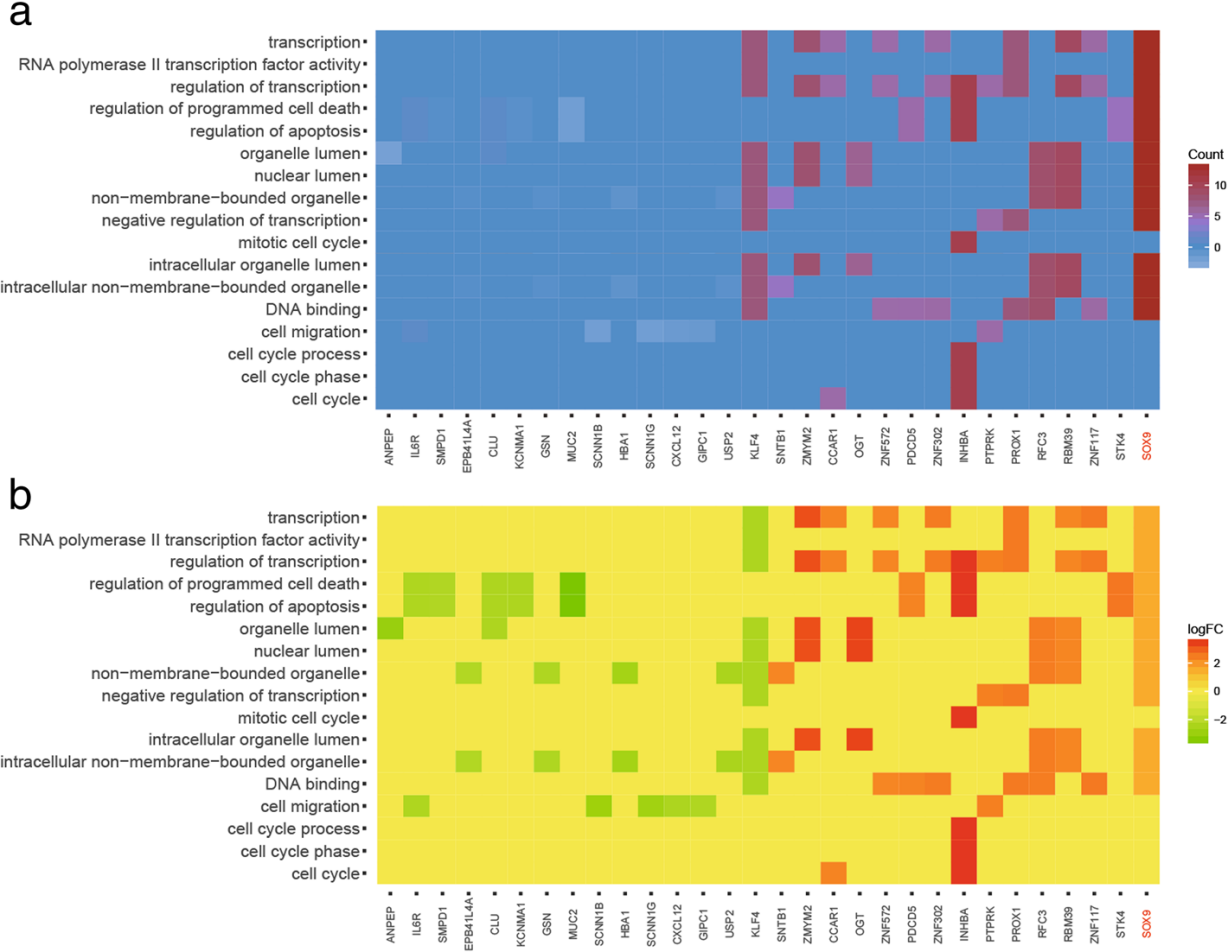
GO analysis and gene enrichment **a** Heat map of GO analysis arranged by count value. **b** Heat map of GO analysis arranged by logFC value

According to the volcano plot, 111 lncRNAs and 3465 mRNAs were found to be up-regulated in colorectal cancer tissues by microarray analysis (Fig. 1a and c). 16 lncRNAs and 18 mRNAs with the largest differences were exhibited in the heat map (Fig. 1b and d). Among them, the expression of lncRNA MALAT1 in cancerous tissues was increased to 2.16 folds compared with that of paracancerous tissues, and the expression of *SOX9* was 1.86 folds greater than that of paracancerous tissues (Fig. 1b and d). Heat map of GO analysis showed the result of gene enrichment in different GO terms. *SOX9* was participated in biological processes like transcription, cell death, apoptosis, migration and so on (Fig. 2a-b). After microarray analysis, a series of differential lncRNAs and genes including MALAT1/SOX9 had been screened out. Then, bioinformatics prediction was performed and we discovered miR-145 was the target of both MALAT1 and SOX9. Among the differential lncRNAs and genes which could establish lncRNA-miRNA-mRNA regulatory axis, MALAT1 and SOX9 were found to be close to colorectal cancer progress through references. Therefore, MALAT1 and *SOX9* were selected as our subsequent study objects.

### Expressions of MALAT1, *SOX9* increased while miR-145 decreased in colorectal cancer tissues

**Fig. 3:**
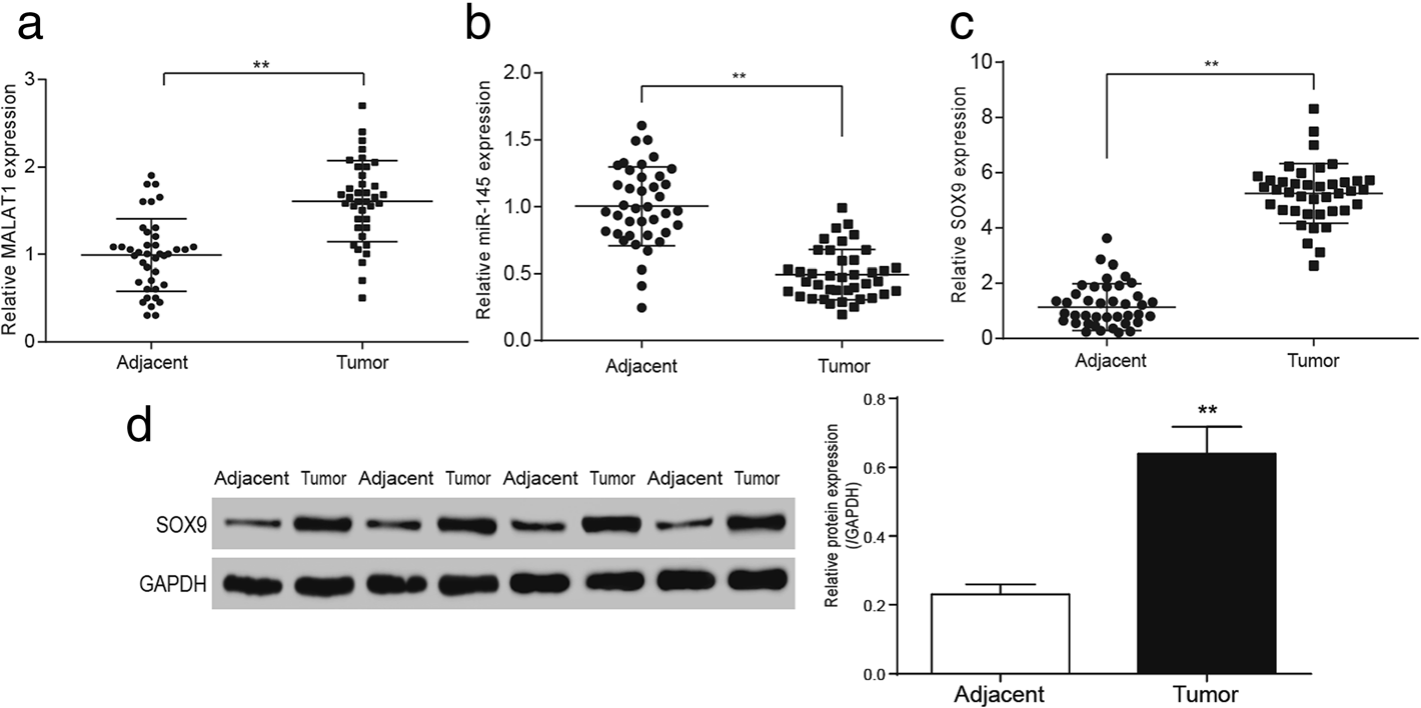
MALAT1 and *SOX9* were highly expressed in colorectal cancer tissues while miR-145 was low expressed **a** The relative MALAT1 expression in colorectal cancer tissues and adjacent tissues. **b** the expression of miR-145 in colorectal cancer tissues and adjacent tissues. **c** qRT-PCR detected the expression of *SOX9* in colorectal cancer tissues and adjacent tissues. **d** western blot detected the expression of *SOX9* in colorectal cancer tissues and adjacent tissues. ^**^P < 0.01, compared with “Adjacent” group

MiR-145, which had targeted relationships with MALAT1 and *SOX9,* was found by miRCode and Targetscan. According to Fig. 3a, the expression of MALAT1 in cancerous tissues significantly increased compared with that of adjacent tissues in the 40 cases of colorectal cancerous tissues, and the difference was statistical (*P* < 0.01). The results of qRT-PCR displayed that miR-145 was low expressed in colorectal cancerous compared with that of adjacent tissues (Fig. 3b, *P* < 0.01). *SOX9* was in a high expression in colorectal cancer compared with that of adjacent tissues (Fig. 3c, *P* < 0.01). Expressions of *SOX9* in colorectal cancerous tissues and paracarcinoma tissues were detected by western blot, the results suggested that the expression of *SOX9* in colorectal cancer tissues was significantly increased than that of adjacent tissues (Fig. 3d, *P* < 0.01). The correlation between MALAT1 / *SOX9* and clinicopathologic characteristic of patients was showed in Table 2. Chi-square test demonstrated that MALAT1 / *SOX9* had a close relation with disease progress like cell invasion and metastasis.

**Table 2:** Correlation between MALAT1/SOX9 expression and clinicopathologic characteristics in 40 cases of colorectal cancer tissues

Characteristics total cases	N of cases 40	MALAT1 expression		*P* value^a^	SOX9 expression		*P* value^a^
		Low	High		Low	High	
Age (years)				0.5092			0.7471
≤ 60	24	9	15		11	13	
> 60	16	8	8		6	10	
Gender				0.5306			0.1074
Male	15	5	10		9	6	
Female	25	12	13		8	17	
AJCC stage				0.012^*^			0.0038^**^
I	10	8	2		8	2	
II	10	5	5		4	6	
III	10	3	7		5	5	
IV	10	1	9		0	10	
Tumor size				0.7471			0.1043
≤ 3 cm	24	11	13		13	11	
> 3 cm	16	6	10		4	12	
Vascular invasion				0.0172^*^			0.0921
Yes	14	2	12		3	11	
No	26	15	11		14	12	
Distant metastasis				0.0786			0.0008^***^
M_0_	29	15	14		17	12	
M_1_	11	2	9		0	11	

*AJCC* American joint committee on cancer

^*^P < 0.05, ^**^P < 0.01, ^***^P < 0.001

^a^Chi-square test

### MiR-145 was the target of MALAT1

**Fig. 4:**
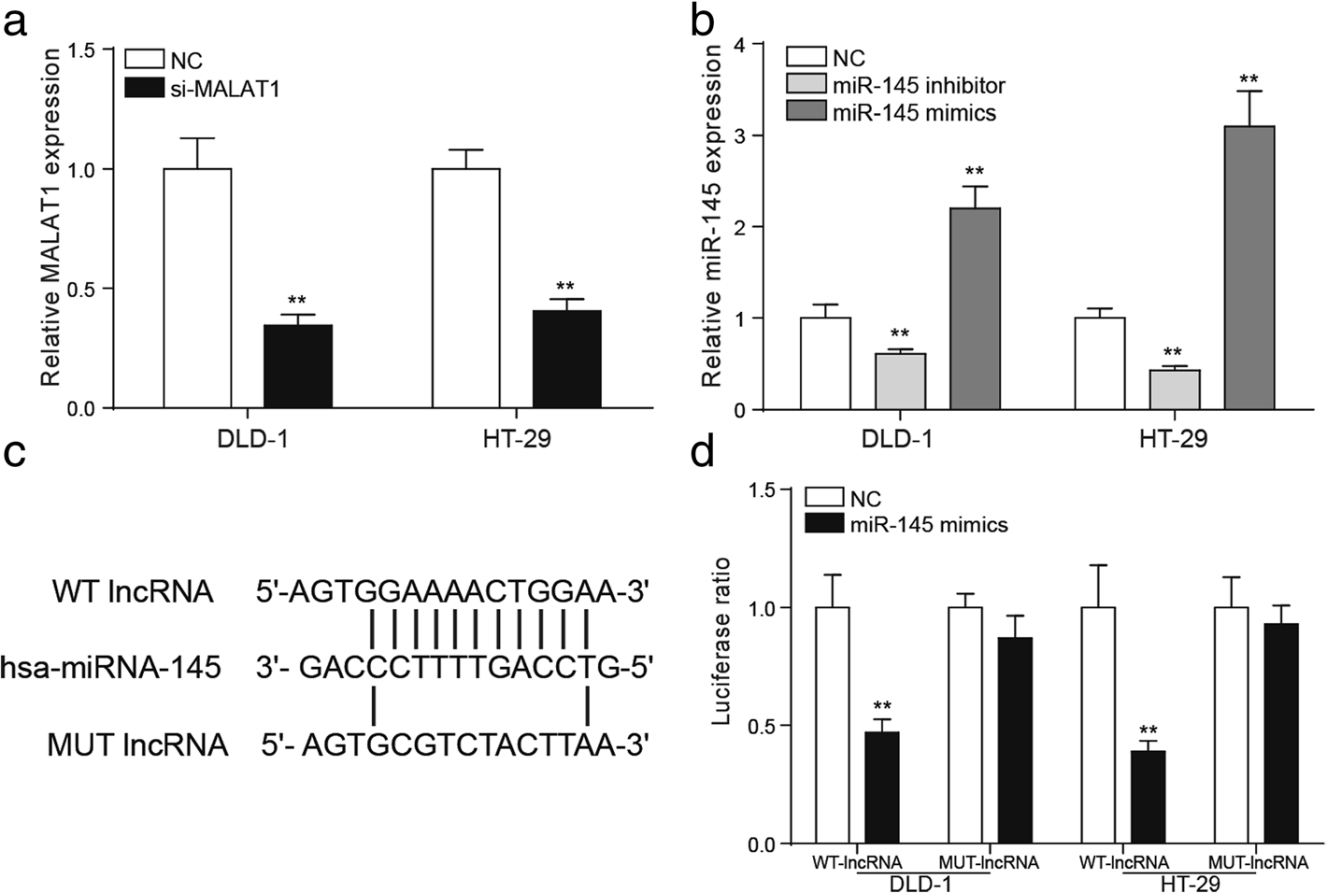
MiR-145 was the target of MALAT1 **a** the relative expression level of MALAT1 after the transfection of si-MALAT1. **b** the relative expression level of miR-145 after the transfection of miR-145 inhibitor or miR-145 mimics. **c** the binding sites of lncRNA and miRNA. **d** it was verified by the dual luciferase reporter that miR-145 was the target gene of MALAT1. ^**^P < 0.01, compared with NC group

QRT-PCR was applied to detect the transfection efficiencies of si-MALAT1, miR-145 inhibitor and miR-145 mimics. The results of qRT-PCR showed that MALAT1 was significantly down regulated in DLD-1 and HT-29 cells after the transfection of si-MALAT1 (Fig. 4a, *P* < 0.01). The expression of miR-145 in DLD-1 and HT-29 cells changed conspicuously after the addition of miR-145 inhibitor of miR-145 mimics (Fig. 4b, *P* < 0.01). The bioinformatics method was used to predict that miR-145 might be the target gene of MALAT1 and the binding site was shown (Fig. 4c). In consideration of structure and cytoplasmic distribution of MALAT1 in colorectal cancer cell, we hypothesized that MALAT1 may function as a competing endogenous RNA to miR-145. Dual-luciferase reporter assay was conducted to detect the targeted relationship between miR-145 and MALAT1. In the mutant group, the fluorescence signal intensity of the NC group and miR-145mimics group was comparative. In the wild-type group, the fluorescence signal intensity of miR-145 mimics group was strikingly lower than that in the NC group (Fig. 4d, *P* < 0.01), indicating that miR-145 was the target of MALAT1.

### MALAT1 accelerated the cell growth, invasion and migration of colorectal cancer cells through down-regulating miR-145

**Fig. 5:**
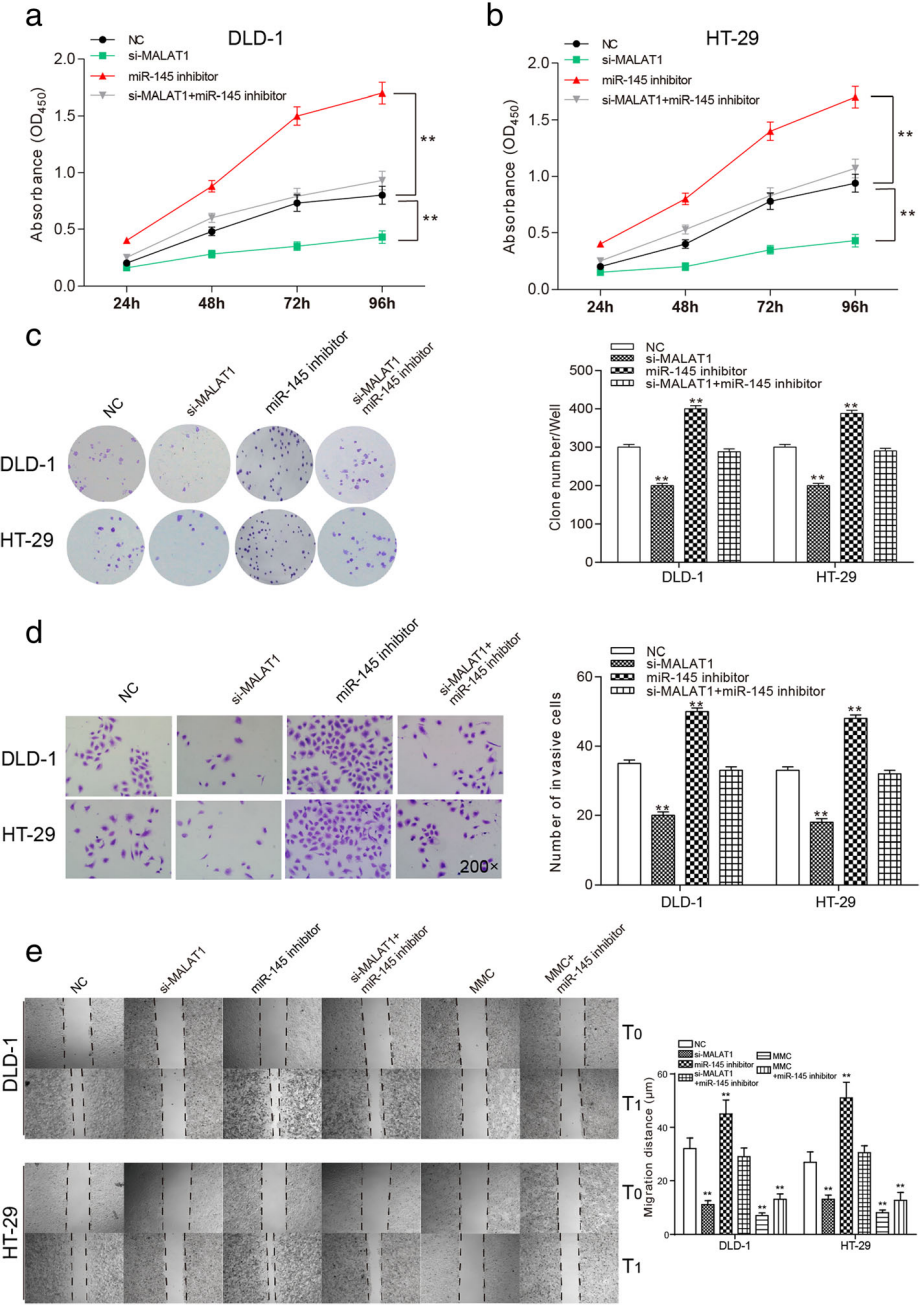
The effects of MALAT1 on proliferation, invasion and migration of colorectal cancer cells through regulating miR-145 **a** and **b** cell proliferation of DLD-1 and HT-29, cell proliferation of si-MALAT1 group was the lowest, NC group and si-MALAT1 + miR-145 inhibitor group was comparative and miR-145 inhibitor group was the highest. **c** colony formation of DLD-1 and HT-29 cells, the number of clone cells was the least in si-MALAT1 group. The miR-145 inhibitor group had the most clone cells. The number of clone cells was comparative in si-MALAT1 + miR-145 inhibitor group and NC group. **d** invasion assay of DLD-1 and HT-29 cells. **e** wound healing assay of DLD-1 and HT-29 cells. ^**^P < 0.01, compared with NC group

The OD value of si-MALAT1 cells was the least in the two types of cell lines. The OD value of cells transfected with miR-145 inhibitor was the largest. The proliferation ability of si-MALAT1 + miR-145 inhibitor group and NC group was comparative (Fig. 5a-b, *P* < 0.01). The number of clone cells was the fewest in si-MALAT1 group. The miR-145 inhibitor group had the most clone cells. The number of clone cells was comparative in si-MALAT1 + miR-145 inhibitor group and the NC group (Fig. 5c, *P* < 0.01). The invasive cells were the least in si-MALAT1 group, si-MALAT1 + miR-145 inhibitor group and NC group took the second place. MiR-145 inhibitor group had the most invasive cells (Fig. 5d, *P* < 0.01). According to Fig. 5e, the migration distance was the shortest in si-MALAT1 group while the longest in miR-145 inhibitor group (*P* < 0.01). Cell migration was significantly suppressed in MCC group and the condition was not changed conspicuously with the addition of miR-145 inhibitor. But there was a big difference between miR-145 inhibitor group and MMC group. No notable difference was revealed between si-MALAT1 + miR-145 inhibitor group and the NC group. These results manifested that low-expression of MALAT1 inhibited proliferation, invasion and migration of colorectal cancer cells.

### Knockdown of MALAT1 induced cell cycle arrest at G1 phase and apoptosis of colorectal cancer cells through up-regulating miR-145

**Fig. 6:**
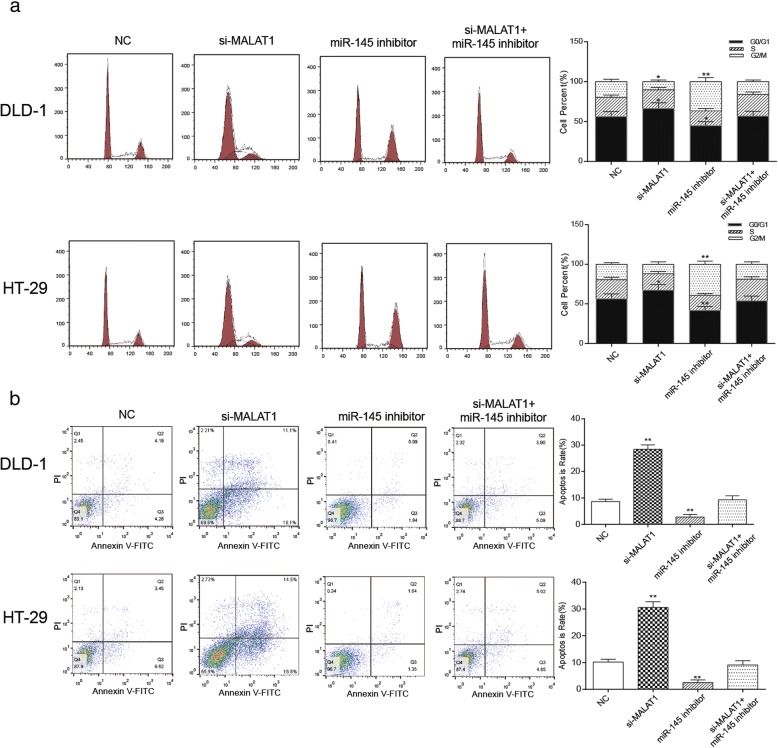
The effects of MALAT1 on cell cycle and apoptosis of cancer cells through regulating miR-145 **a** the experimental results of cell cycle of the two types of cells under different interference conditions, si-MALAT1 group had the most cells in G1 phase and the least cells in G2 phase. The number of cells in G2 phase was the most in miR-145 inhibitor group **b** experimental results of apoptosis of two types of cells under different interference conditions, the apoptosis ratio of si-MALAT1 group was the highest, and the miR-145 inhibitor group was the lowest, the si-MALAT1 + miR-145 inhibitor group and the NC group was comparative. ^*^P < 0.05, ^**^P < 0.01, compared with NC group

In DLD-1 and HT-29, the number of cancer cells in the G1 phase increased in the si-MALAT1 group while decreased in miR-145 inhibitor group in contrast to the NC group. The cell number of in the G1 phase in the NC group and the si-MALAT1 + miR-145 inhibitor group was comparable. In addition, cancer cells in the G2 phase increased in miR-145 inhibitor group and decreased in the si-MALAT1 group compared with the NC group (Fig. 6a, *P* < 0.05). These results indicated that down-regulation of MALAT1 induced the G1 phase arrest of colorectal cancer cells. As shown in Fig. 6b, compared with the NC group, the apoptosis ratio was significantly rose in the si-MALAT1 group but obviously reduced in miR-145 inhibitor group (*P* < 0.01). The apoptosis rate of the si-MALAT1 + miR-145 inhibitor group had no significant difference from that of the NC group. These indicated that the knockdown of MALAT1 could promote apoptosis of cells, while the down-regulation of miR-145 inhibited cell apoptosis.

### *SOX9* was the target gene of miR-145

**Fig. 7:**
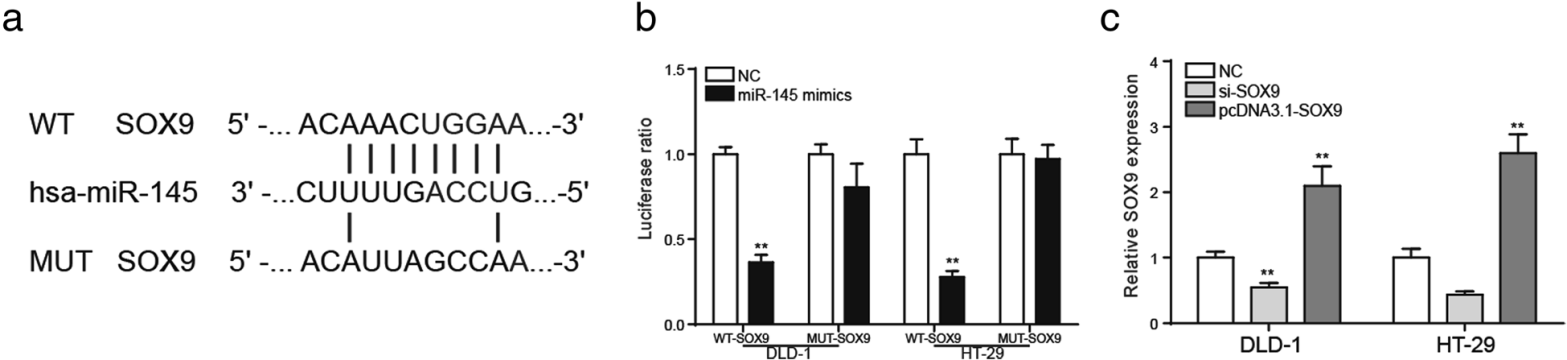
*SOX9* was the target gene of miR-145. **a** the binding sites of miR-145 and *SOX9*. **b** results of *SOX9* enrichment, *SOX9* was the target gene of miR-145. **c** Transfection efficiency test after the transfection of si-*SOX9* or pcDNA3.1-*SOX9*. ^**^P < 0.01, compared with NC group

The bioinformatics method was used to predict that miR-145 might be the target gene of MALAT1 and the binding site was shown in Fig. 7a. According to Fig. 7b, in the mutant group, the luciferase ratio of miR-145 mimics group was comparative to that of NC group (*P* < 0.01). But in wild-type group, it was lower in miR-145 mimics group than in the NC group. It uncovered that miR-145 had a targeted relationship with *SOX9*. The qRT-PCR analysis showed that *SOX9* expression changed conspicuously after the transfection of si-*SOX9* or pcDNA3.1-*SOX9* (Fig. 7c, *P* < 0.01).

### *SOX9* could promote proliferation, invasion and migration of colorectal cancer cells

**Fig. 8:**
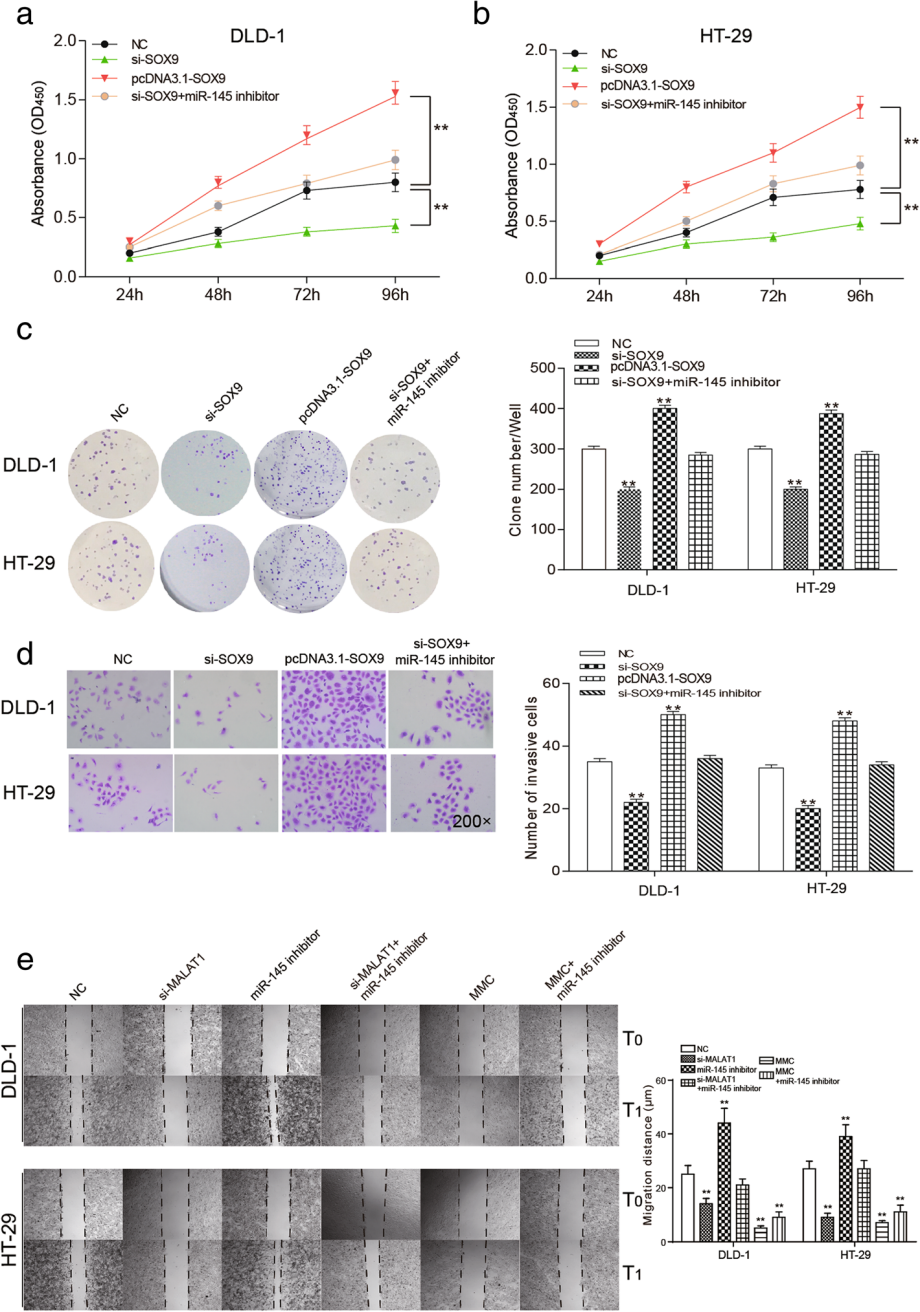
Effects of *SOX9* on proliferation, invasion and migration of colorectal cancer cells **a** and **b** the cell proliferation of colorectal cancer cell lines DLD-1 and HT-29 transfected with different interference sequences, cell proliferation was the weakest in si-*SOX9* group, and pcDNA3.1-*SOX9* group had the strongest cell proliferation ability compared with NC group. The cell proliferation of si-*SOX9* + miR-145 inhibitor group and NC group was comparative. **c** cell colony formation of colorectal cancer, the formation rate of colorectal cancer cells in the si-*SOX9* group was remarkably slower than that of the NC group. The cell formation rate was significantly higher in pcDNA3.1-*SOX9* group than that of NC group, and the cell formation in si-*SOX9* + miR-145 inhibitor group and NC group had no significant difference. **d** invasion assays of two kinds of cancer cells under different interference conditions. **e** the scratch wound healing assays of two kinds of cancer cells under different interference conditions. ^**^P < 0.01, compared with NC group

After 96 h, OD value was the lowest in si-*SOX9* group, and highest in pcDNA3.1-*SOX9* group. The OD value of si-*SOX9* + miR-145 inhibitor group showed no significant change compared with the NC group, suggesting that the down-regulation of *SOX9* inhibited the proliferation of colorectal cancer cells (Fig. 8a-b, *P* < 0.01). Similarly, in the two groups of cell lines in the colony formation assay, the clone number of colorectal cancer cells with the knockdown of *SOX9* was significantly smaller than that of the NC group. After the over-expression of *SOX9*, the clone number was significantly improved. The clone number in si-*SOX9* + miR-145 inhibitor group and NC group had no significant difference (Fig. 8c, *P* < 0.01). Twenty-four hours after transfection, the number of invasive cells and migration distance significantly decreased in si-*SOX9* group but increased in pcDNA3.1-*SOX9* group compared with that of the NC group. Migration distance and number of invasion cells were undifferentiated in si-*SOX9* + miR-145 inhibitor group and the NC group (Fig. 8d-e, *P* < 0.01). There was a big difference between miR-145 inhibitor group and MMC group. These results suggested that down-regulation of *SOX9* could effectively suppress proliferation, invasion and migration of colorectal cancer cells.

### Knockdown of *SOX9* could induce cell cycle arrest at G1 phase and apoptosis of colorectal cancer cells

**Fig. 9:**
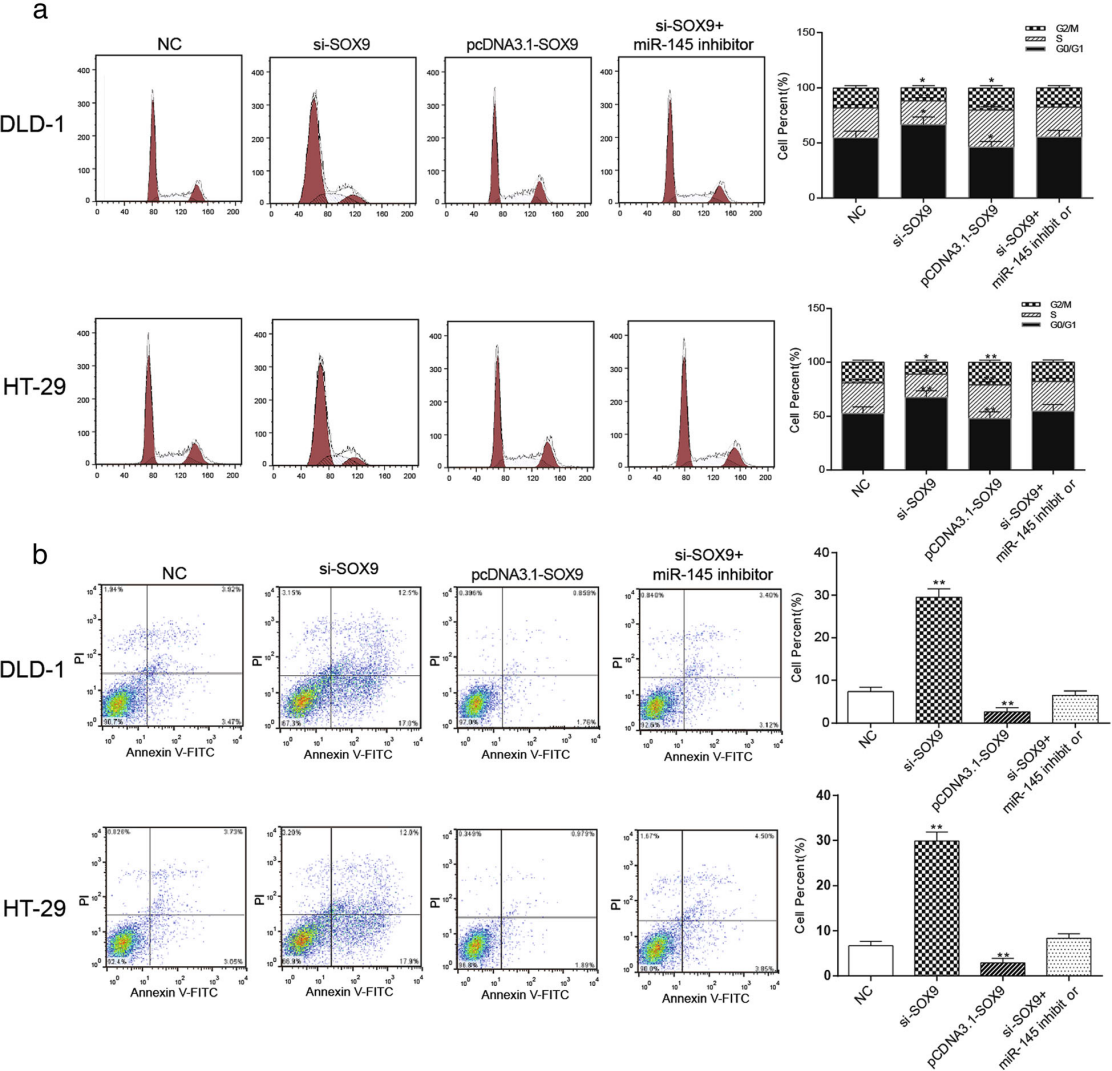
Effects of *SOX9* on cycle and apoptosis of colorectal cancer cells **a** the cell cycle was assessed by flow cytometry, the number of cells in G1 phase was the most in the si-*SOX9* group and was the least in pcDNA3.1-*SOX9* group, the si-*SOX9* + miR-145 inhibitor group was comparative to the NC group. **b** The apoptosis rate which measured by flow cytometry of pcDNA3.1-*SOX9* group was the highest, si-*SOX9* + miR-145 inhibitor group and NC group took the second place, and si-*SOX9* group had the lowest apoptosis rate. ^*^P < 0.05, ^**^P < 0.01, compared with NC group

As Fig. 9a revealed, the number of cells in G1 phase was obviously increased in the si-*SOX9* group, remarkably decreased in pcDNA3.1-*SOX9* group and didn’t change much in si-*SOX9* + miR-145 inhibitor group compared with the NC group (*P* < 0.05). The apoptosis rate of pcDNA3.1-*SOX9* group was significantly higher than that of the NC group, si-*SOX9* + miR-145 inhibitor group and NC group took the second place, and si-*SOX9* group had the lowest apoptosis rate (Fig. 9b, *P* < 0.01). We might draw the conclusion that down-regulation of *SOX9* promoted cell apoptosis and induced G1 cell cycle arrest of colorectal cancer.

### Down-regulating MALAT1 could prevent cell proliferation of colorectal cancer by up-regulating miR-145 in vivo

**Fig. 10:**
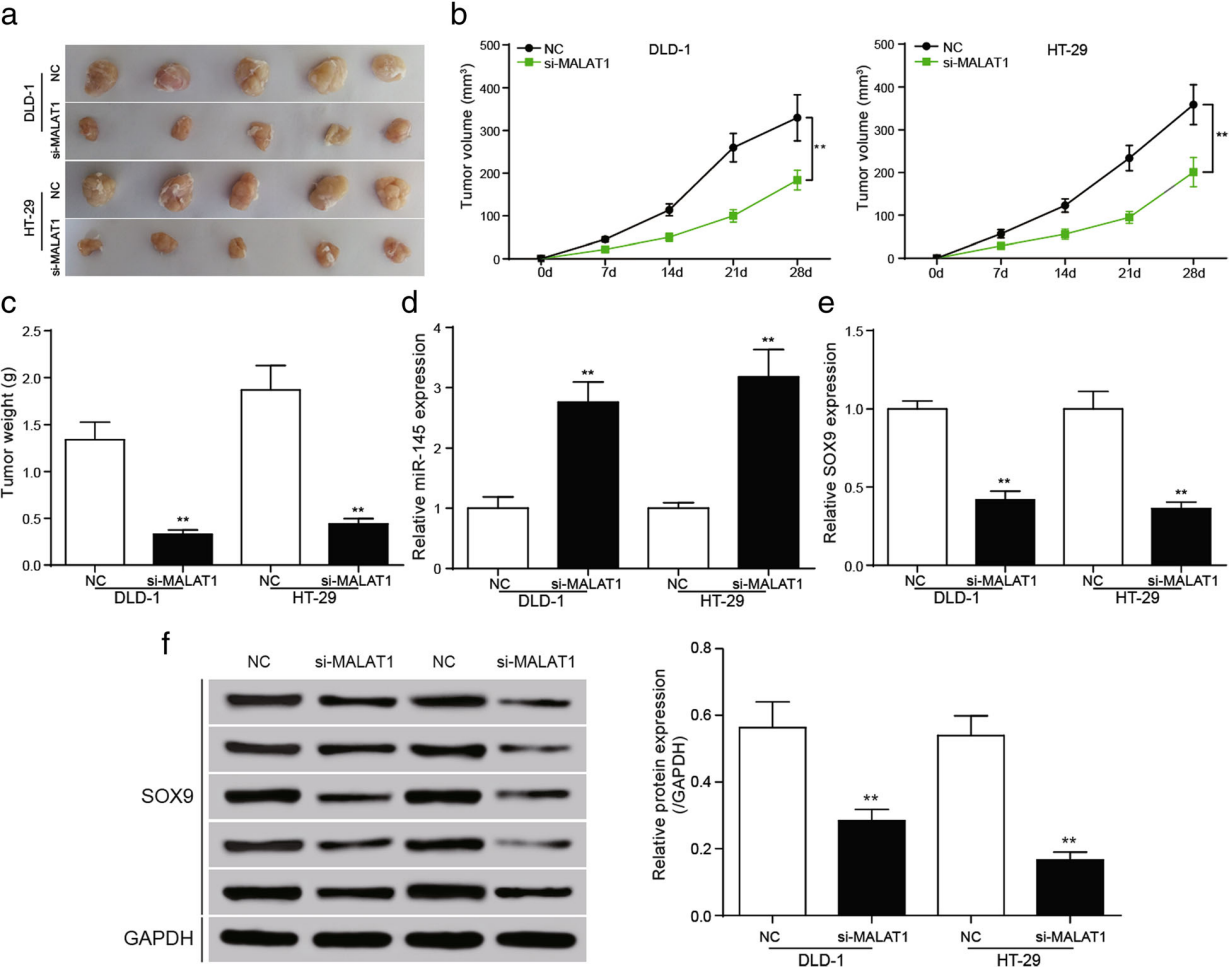
Down-regulation of *SOX9* inhibited the growth of tumor in vivo **a** the tumor specimen figure of nude mice. **b** statistics of tumor volume, the growth of si-MALAT1 group and si-*SOX9* group was the slowest, and the volume was the smallest. The tumor volume of NC group and si-*SOX9* + miR-145 inhibitor group, si-MALAT1 + pCDNA3-*SOX9* group was comparative. pcDNA3.1-*SOX9* group had the largest tumor volume. **c** Statistical results showed that tumor weight in nude mice, the tumor weight was significantly reduced in si-MALAT1 group. **d** The relative miR-145 expression was conspicuously increased in si-MALAT1 group in tumor tissues. **e**, **f** The qRT-PCR and western blot showed *SOX9* expression was significantly decreased in si-MALAT1 group in tumor tissues. ^**^P < 0.01, compared with NC group

The nude mice were subcutaneously injected with DLD-1 and HT-29 cells that transfected with si-NC and si-MALAT1. The tumor size was smaller in si-MALAT1 group. And in this group, the tumor volume were lower than that in the NC group (Fig. 10a and b, *P* < 0.01). The weight of the tumor had the similar trend of the NC group (Fig. 10a and c, *P* < 0.01). We could see that in si-MALAT1 group, the expression of miR-145 was higher and *SOX9* was lower than NC group, further verifying the knockdown of MALAT1 inhibited the growth of colorectal cancer in vivo by up-regulating miR-145 and down-regulating *SOX9* (Fig. 10d-f, *P* < 0.01)*.*

## Discussion

In this present study, we noted that significantly up-regulated MALAT1 expression was observed in colorectal cancer tissues and cells. Moreover, over-expression of MALAT1 enhanced the cell growth of colorectal cancer cells, whereas miR-145 showed the opposite effect through repressing *SOX9* expression. The down-regulation of miR-145 by competing endogenous RNA MALAT1 led to up-regulation of *SOX9*. Our results showed that MALAT1 might play an essential role via the miR-145-*SOX9*-mediated pathway in the development of colorectal cancer.

MALAT1, which is also known as PRO2853, HCN, NCRNA00047, and NEAT2, is one of the first lncRNAs described to play a significant role in cancer metastasis (Ji et al. 2003). We found that MALAT1 up-regulated in colorectal cancerous cells and tissues, which were coincident with previously reports. MALAT-1 has been demonstrated to be up-regulated in many types of cancer, such as prostate cancer (Ren et al. 2013), breast cancer (Jadaliha et al. 2016) and monocytic leukemia (Huang et al. 2017). In this study, endogenous competition function of MALAT1 was verified through down-stream miR-145 expression. Silencing MALAT1 significantly inhibited cell growth, migration and invasion. Consistent with results of our experiments, previous studies also suggested that higher expression of MALAT1 significantly correlated with metastasis in patients with cancers through the transcriptional and post-transcriptional modulation. For instance, MALAT1 RNA promoted migration and tumor growth of non-small cell lung cancer (Schmidt et al. 2011). Additionally, MALAT1 has been demonstrated to enhance cell motility of lung adenocarcinoma cells (Tano et al. 2010) and promote the migration and invasion of gastric cancer cells via EMT (Chen et al. 2017). MALAT1 also played an important role in cell cycle and cell apoptosis (Gutschner et al. 2013). It was illustrated that down-regulation of MALAT1 significantly increased G1 phase and decreased S phase in cervical cancer, and resulted in cell apoptosis increased obviously (Guo et al. 2010). Similar to these findings, our study also identified that knockdown of MALAT1 promoted cell cycle arrest and cell apoptosis.

Up to now, studies on tumor metastasis have reported that miRNAs play a role in regulating oncogenes and tumor suppressors (Dalmay and Edwards 2006; Lim et al. 2005). There have been several studies examining the expression patterns of miRNAs and its role in the progression of cancers (Asangani et al. 2008). In the present study, miR-145 expression was significantly decreased in colorectal cancer tissues. This study further demonstrated that down-regulation of miR-145 promoted the development of colorectal cancer, indicating a potential role for miR-145 in tumor invasion and metastasis. Consistent with our results, reports showed that in colorectal cancer, miR-145 inhibited tumor growth and metastasis via regulating fascin-1 (Feng et al. 2014). Besides, miR-145 was identified in clinical colorectal cancer samples, which showed that miR-145 weakened the migrating and invading ability of cancer cells by targeting an ETS-related gene (Li et al. 2016). Hence, the specific biological function of miR-145 in the process of colorectal cancer metastasis remained to be importantly and necessarily studied. All these above can provide a powerful reference for clinical treatment.

*SOX9*, one target of miR-145, has been reported up-regulated in many carcinomas and has been regarded to be an important oncogene which promotes migration (Liu et al. 2017; Xiong et al. 2017) found that *SOX9* functioned as a tumor promotion in hepatocellular carcinoma partially induced by miR-138 (Liu et al. 2016b). Contrary to these reports, in our study, miR-145 led to down-regulation of *SOX9*, which resulted in the suppression of colorectal cancerous cell growth, migration and invasion. We speculate that this inconsistency may result from the insufficient number of cases or that *SOX9* may have a special role in colorectal cancer.

## Conclusions

To sum up, our study tested and verified that MALAT1 was highly expressed in colorectal cancer tissues and cells. MALAT1 regulated miR-145 expression as a competing endogenous RNA and down-regulation of MALAT1 suppressed proliferation and invasion, promoted cell cycle G1 phase and apoptosis of cancerous cells by increasing the expression of miR-145 and decreasing *SOX9* expression. Therefore, MALAT1, miR-145 and *SOX9* are indicated to be novel promising candidates for developing effective therapeutic strategies for colorectal cancer.

## Abbreviations

ANOVA: Analysis of variance
ECL: Enhanced chemiluminescence
GEO: Gene Expression Omnibus
miRNAs: MicroRNAs
PE: The efficiency of plating
SF: Survival rate
TBST: Tris-buffered saline with Tween
